# Whole Genome Sequence Data From Captive Baboons Implicate *RBFOX1* in Epileptic Seizure Risk

**DOI:** 10.3389/fgene.2021.714282

**Published:** 2021-08-20

**Authors:** Mark Z. Kos, Melanie A. Carless, Lucy Blondell, M. Michelle Leland, Koyle D. Knape, Harald H. H. Göring, Charles Ákos Szabó

**Affiliations:** ^1^Department of Human Genetics, South Texas Diabetes and Obesity Institute, University of Texas Rio Grande Valley School of Medicine, Edinburg, TX, United States; ^2^Department of Biology, The University of Texas at San Antonio, San Antonio, TX, United States; ^3^Brain Health Consortium, The University of Texas at San Antonio, San Antonio, TX, United States; ^4^Laboratory Animal Research, UT Health San Antonio, San Antonio, TX, United States; ^5^Department of Neurology, UT Health San Antonio, San Antonio, TX, United States; ^6^South Texas Comprehensive Epilepsy Center, San Antonio, TX, United States

**Keywords:** whole-genome sequence, association test, *Papio*, baboon, idiopathic generalized epilepsy, epilepsy, genetic generalized epilepsy, *RBFOX1*

## Abstract

In this study, we investigate the genetic determinants that underlie epilepsy in a captive baboon pedigree and evaluate the potential suitability of this non-human primate model for understanding the genetic etiology of human epilepsy. Archived whole-genome sequence data were analyzed using both a candidate gene approach that targeted variants in baboon homologs of 19 genes (*n* = 20,881 SNPs) previously implicated in genetic generalized epilepsy (GGE) and a more agnostic approach that examined protein-altering mutations genome-wide as assessed by snpEff (*n* = 36,169). Measured genotype association tests for baboon cases of epileptic seizure were performed using SOLAR, as well as gene set enrichment analyses (GSEA) and protein–protein interaction (PPI) network construction of top association hits genome-wide *(p* < 0.01; *n* = 441 genes). The maximum likelihood estimate of heritability for epileptic seizure in the pedigreed baboon sample is 0.76 (SE = 0.77; *p* = 0.07). Among candidate genes for GGE, a significant association was detected for an intronic SNP in *RBFOX1 (p* = 5.92 × 10^–6^; adjusted *p* = 0.016). For protein-altering variants, no genome-wide significant results were observed for epilepsy status. However, GSEA revealed significant positive enrichment for genes involved in the extracellular matrix structure (ECM; FDR = 0.0072) and collagen formation (FDR = 0.017), which was reflected in a major PPI network cluster. This preliminary study highlights the potential role of RBFOX1 in the epileptic baboon, a protein involved in transcriptomic regulation of multiple epilepsy candidate genes in humans and itself previously implicated in human epilepsy, both focal and generalized. Moreover, protein-damaging variants from across the genome exhibit a pattern of association that links collagen-containing ECM to epilepsy risk. These findings suggest a shared genetic etiology between baboon and human forms of GGE and lay the foundation for follow-up research.

## Introduction

Epilepsy is a chronic, highly heterogeneous neurological disorder, with recurrent seizures being the diagnostic hallmark. Genetic generalized epilepsies (GGEs) account for at least 40% of United States cases, (Durón et al., 2005) with heritability estimates from twin studies ranging from 25 to 69% (Kjeldsen et al., 2001). With the rise of high-throughput genomic sequencing, a multitude of epilepsy-associated genetic variants have been identified in recent years, including rare variants, such as recurrent microdeletions of large effect (Epi 4K Consortium and Epilepsy Phenome/Genome Project, 2017). Currently, epilepsies are suspected of having complex polygenic architectures, involving variants of many genes from across the frequency spectrum. However, segregation analyses in some families have shown that rare pathogenic variants convey most of the risk, (Myers and Mefford, 2016) with a potentially important role played by *de novo* mutagenesis, as established in patients with focal and generalized epilepsies as well as epileptic encephalopathies (Jones et al., 2011).

Toward elucidating the various genes and molecular mechanisms underlying epileptogenesis and seizure generation, animal models have played a critical role, as clinical studies of human patients preclude the possibility of invasive, and often more insightful, methods. For decades now, rodents have been the most common animal models used in epilepsy research, contributing greatly toward our understanding of acquired epilepsy and acutely induced seizure events. For chronic phenotypes that more closely resemble human epilepsy, a number of well-described mouse models have been developed that exhibit spontaneous absence-like seizures with spike-wave EEG discharges analogous to GGE, but with important drawbacks, most notably their monogenic basis and other neurological abnormalities. More promising are two rat models with presumed polygenic inheritance, Genetic Absence Rats from Strasbourg (GAERS) and Wistar Albino Glaxo rat from Rijswijk (WAG/Rij), (Gauguier et al., 2004; Rudolf et al., 2004) that have shown electroclinical and pathophysiological similarities to human GGE with absence epilepsy. Despite these successes, translatability of these models is limited due to differences in neurodevelopment and the natural evolutionary histories of the species, as well as response to treatment, (Löscher, 2017) suggesting the need for alternate animal models that can further characterize the complex genetic architecture of GGE in humans and better identify new etiological pathways with novel therapeutic targets.

Baboons are suitable genetic models for humans, as their genomes are more similar to humans than those of less closely related model organisms, and share neuroanatomical, biochemical, and physiological features stemming from their close phylogenetic relationship. Baboons have been used as animal models in a broad range of human diseases, including diabetes, heart disease, osteoporosis, and chronic infectious diseases (Rogers and Hixson, 1997). Baboons also represent a natural model for GGE (Szabó et al., 2012, 2013; Croll et al., 2019). For the pedigreed baboon colony housed at the Southwest National Primate Research Center (SNPRC; San Antonio, TX, United States), absence, myoclonic, and generalized tonic–clonic seizures have been reported to occur spontaneously or triggered by intermittent light stimulation, with electroclinical findings suggesting strong similarities with human epilepsy, in particular juvenile myoclonic epilepsy (Szabó et al., 2013). In a preliminary analysis of 1,400 animals, (Croll et al., 2019) we previously estimated moderate heritabilities for seizures (*h*^2^ = 0.33, *p* < 1 × 10^–7^) and interictal epileptic discharges (*h*^2^ = 0.19, *p* < 0.002), although the underlying genetic variants are unknown. With high-throughput whole-genome sequencing (WGS) now performed on a subset of these animals and the potential enrichment of rare pathogenic variants within large multigenerational pedigrees that improve statistical power for detecting their risk effects, the SNPRC baboon colony represents a largely untapped genetic resource for epilepsy research (Jun et al., 2018).

This study provides an initial assessment of the baboon as a genetic model for epilepsy. Archived WGS data from the SNPRC colony were tested for genetic associations with epileptic seizure, targeting single nucleotide polymorphisms (SNPs) within GGE-associated genes, as well as protein-altering variants genome-wide. Based on these results, as well as pathway enrichment analyses of top association hits, a significant association was observed for a common variant in the gene *RBFOX1* (RNA Binding Fox-1 Homolog 1), providing insight into the etiological underpinnings of the disorder.

## Materials and Methods

### Baboon Colony

There are currently about 1,000 baboons in the pedigreed colony housed at the SNPRC, representing a mixture of species, with extensive veterinary records generated since 1980. The animals have been treated in strict accordance with United States Public Health Service policy, (Institute for Laboratory Animal Research, 1996) with regular inspections by the United States Department of Agriculture (latest inspection in 2019). This study was approved by the IACUC of the University of Texas Health Science Center at San Antonio and the Texas Biomedical Research Institute.

We performed a retrospective survey of seizures from veterinary records available for 1,528 baboons, both living and deceased. This included both witnessed seizures, either spontaneous or provoked by ketamine administration or handling, and suspected seizures, assessed by treatments for head injuries, in particular craniofacial trauma that are often related to falls during seizures, as well as peri-ictal behaviors such as confusion and lethargy. As previously reported, (Szabó et al., 2012) a total of 3,389 seizure events were detected in the records of 1,098 baboons, of which 1,537 (45%) were witnessed in 404 animals, with 1,267 (82%) being unprovoked. For this study, archived WGS data were available for 90 baboons with clinical histories [*Papio hamadryas anubis (P.h. anubis)* and *P.h. anubis/cynocephalus* hybrids], including scalp EEG recordings of interictal epileptic discharges for 53 of these animals (Szabó et al., 2013). Diagnosis of epilepsy was adapted from criteria established by the International League Against Epilepsy (ILAE), (Fisher et al., 2014) including: (1) evidence of at least one witnessed seizure or craniofacial trauma with a diagnostic EEG; (2) two or more witnessed seizures or craniofacial trauma without a diagnostic EEG; (3) or recording of seizures, spontaneous or photoepileptic, by video-EEG (Szabó et al., 2013, 2014). Baboons without a history of witnessed seizures and/or craniofacial trauma and normal EEG measures constituted our control group. Using these criteria, 42 baboons were diagnosed with epilepsy, 19 as healthy controls, and 29 as indeterminate (single witnessed seizure or acute craniofacial trauma and without a diagnostic EEG).

### Processing of Archived WGS Data

Archived WGS data for the SNPRC baboon colony are available in the NCBI Sequence Read Archive (SRA) repository. These data have been submitted under BioProject PRJNA433868, described as a “mixture of low-coverage and high-coverage whole-genome sequence data” generated on Illumina HiSeq X Ten and HiSeq 2500 instruments. Using a submission date cut-off of 8/2/2018, a total of 791 WGS runs representing 788 different baboons were downloaded as compressed SRA-formatted files onto the STDOI Genomics Computing Center cluster at the University of Texas Rio Grande Valley using the SRA FTP platform (command *prefetch*, followed by *fastq-dump*), representing approximately 11 TB of data.

For the 90 baboons that were assessed for epileptic seizure, FASTQ files were extracted from the compressed WGS files, representing about 6.8 × genomic coverage on average, and then aligned against the reference *P.h. anubis* genome assembly “Panu_3.0” (NCBI BioProject PRJNA54005) with the software BWA v. 0.7.12, (Li and Durbin, 2010) using the faster and more accurate BWA-MEM algorithm (Li, 2013) and compressed into the binary BAM format using the *SamFormatConverter* Java executable in the software package Picard v. 1.121^1^ Using the Picard toolkit, the aligned sequences were then (1) soft-clipped for beyond-end-of-reference alignments with *CleanSam*; (2) sorted based on genomic coordinates with *AddOrReplaceReadGroups*, with meta information added to the reads; (3) tagged for duplicate reads with *MarkDuplicates*; (4) confirmed for their consistency to the SAM format specifications with *ValidateSamFile*; and (5) used to generate BAM index files with *BuildBamIndex* to allow for faster processing of the data downstream.

For the next steps in the WGS pipeline, the GATK genomic analysis toolkit (v. 3.5 and v. 4.1.3.0) (McKenna et al., 2010) was employed as follows: (1) potential mapping errors due to indel events were identified in the tagged BAM files and realigned with *RealignerTargetCreator* and *IndelRealigner*; (2) quality scores for individual base calls were examined and adjusted for systematic technical errors stemming from the original sequencing work using base quality score recalibration (BQSR), a machine-learning approach to empirically build a covariation model of these errors using a set of known variants [in this case, previously published sequence variation of six species of *Papio* baboon and *Theropithecus gelada* (Rogers et al., 2019)]; (3) call SNPs from realigned and recalibrated sequence data *via* local *de novo* assembly using *HaplotypeCaller* (using -ERC GVCF mode); (4) aggregate the resulting 90 GVCF files into multi-sample “datastores” per chromosome using *GenomicsDBImport*; and (5) perform joint genotyping on each datastore with *GenotypeGVCFs*, creating raw SNP VCF files.

Lastly, based on recommendations made by the GATK development team,^2^ low-quality SNPs were excluded from the genotyped VCF files using *SelectVariants* for the following hard thresholds: Phred-scaled quality score (QUAL) < 30; quality score by depth (QD) < 2; symmetric odds ratio (SOR) > 3; measure of strand bias by Fisher’s exact test (FS) > 60; root mean square of mapping quality (MQ) < 40; rank sum test for mapping qualities of reference and alternate allele reads (MQRankSum) < −12.5; rank sum test for relative positioning of reference and alternate alleles within reads (ReadPosRankSum) < −8; and allele frequency (AF) < 0.05.

### Genetic Analyses

The WGS data were examined using both candidate gene and genome-wide approaches to improve the statistical power of the study. For the former, genes implicated in GGE or the synonymous idiopathic generalized epilepsy (IGE) were reviewed as positional candidate genes by searching the PubMed database with the following terms:

(GENE NAME) AND [(“genetic generalized epilepsy”) OR (“idiopathic generalized epilepsy”) OR (“childhood absence epilepsy”) OR (“juvenile absence epilepsy”) OR (“juvenile myoclonic epilepsy”)].

The search was not exhaustive but limited to genes with *a priori* evidence for GGE susceptibility in the databases *Online Mendelian Inheritance in Man* (OMIM) and *EpilepsyGene*, (Ran et al., 2015) including genetic epilepsy with febrile seizures plus, childhood absence epilepsy, juvenile absence epilepsy, and juvenile myoclonic epilepsy. Positional candidate genes were identified as such by evaluating the collective *in silico* evidence yielded by the PubMed search, including the seminal GWAS mega-analysis conducted by ILAE, (The International League Against Epilepsy Consortium on Complex Epilepsies, 2018) with particular emphasis paid to studies that *independently* reported significant associations or familial segregation of gene variants and any of the various forms of GGE (thus disqualifying novel, unreplicated findings). In the end, 19 positional candidate genes for GGE were selected for our study, with each having a homologous gene or homolog in the baboon genome (confirmed using the chain/net alignments for genomes GRCh38 and Panu_3.0 from the UCSC Genome Browser): *BRD2*, *CACNA1A*, *CACNA1H*, *CACNB4*, *CASR*, *CHRNA7*, *EFHC1*, *GABRA1*, *GABRA6*, *GABRB3*, *GABRD*, *GABRG2*, *ICK*, *ME2*, *NIPA2*, *PNPO*, *RBFOX1*, *SCN1A*, and *SLC2A1*.

Heritability (*h*^2^) estimation and genetic association testing were performed using the software SOLAR, (Almasy and Blangero, 1998) based on maximum likelihood variance decomposition. Genetic relationships between the baboons were inferred from a previously reported, single six-generation pedigree comprising 2,455 animals, including 38 cases of epileptic seizure and 16 controls with processed WGS data (total *n* = 54), which were the focus of the genetic analyses. Associations between SNPs and seizure status were computed using measured genotype association (MGA) analysis under a liability threshold in which kinship is treated as a random effect and SNPs as fixed effects, with sex and the first principal component (PC) as covariates. The likelihoods of the MGA models, each maximized for the effects of kinship and SNP genotypes, were compared to null models with SNP effects constrained to zero. For the baboon homologs of the 19 positional candidate genes for GGE, 20,881 SNPs were identified in the WGS data (including variants 5 kb upstream and downstream of the leading and trailing UTR sequences) and tested for association. A more agnostic, genome-wide approach was also taken, in which protein-altering variants of “moderate” impact (e.g., missense variants) and “high” impact (e.g., start and stop codons gained or lost, splice acceptor and donor site mutations) on protein function were tested in a separate run (*n* = 36,169 SNPs). These variants were predicted by the annotation tool SnpEff (Cingolani et al., 2012b) using a SNP prediction library built for assembly Panu_3.0/Panu 4 (gene annotation GTF files downloaded from the UCSC database) and filtered with SnpSift (Cingolani et al., 2012a). Association *p*-values were adjusted for the effective number of independent SNPs tested, which was estimated per chromosome in SOLAR using the Li & Ji method (Li and Ji, 2005) based on the computation of eigenvalues of a genotypic correlation matrix.

For the association results of genome-wide deleterious SNPs, the genes impacted by these variants (and with known human homologs) were tested for enrichment for genes from biological pathways and molecular ontologies using the program Gene Set Enrichment Analysis (GSEA v. 4.1) (Subramanian et al., 2005) for the Molecular Signatures Database (MSigDB v. 7.2). GSEA represents a modified Kolmogorov–Smirnov test, with significance of the unweighted enrichment score established *via* 10,000 permutations. Additionally, protein–protein interaction (PPI) networks were constructed for these results using the online tool STRING v. 11.0, (Szklarczyk et al., 2019) limited to interactions of “high confidence” (score ≥ 0.7) from all available sources, with functional enrichment analysis performed on PPI nodes for the entire network, as well as targeted analysis of the largest observed cluster, for reactome pathways and GO-terms.

### Assessment of Inflation of the Association Test Statistic

Potential inflation of the MGA test statistic was assessed by calculating the genomic inflation factor λ (ratio of the median of the empirically observed distribution of χ^2^ to the expected median of 0.456). This was done for 10 random subsets of 20,881 intragenic SNPs from the processed WGS data (mirroring the number of SNPs tested in the 19 candidate genes). For the MGA model with sex as the sole covariate, some inflation was observed among association results generated for these random subsets, with a mean λ of 1.13 (SD = 0.02), suggesting potential confounding due to population substructure. To investigate this, principal components analysis (PCA) was performed on WGS data from autosomes 1–20 (*n* = 5,542,179 SNPs) with the EIGENSTRAT method, using the statistical package AssocTests v. 1.0-1 (Wang et al., 2020) in R v. 4.0.3. (R Core Team, 2020). A plot of the first two PCs (accounting for 9.0 and 4.6% of the genotypic variation, respectively; see Supplementary Figure 1) revealed stratification within the baboon sample, especially relative to the first PC. Hence, PC1 was included as a covariate in the MGA model and association testing was re-run on the random SNP subsets, effectively controlling the genome-wide inflation, with a mean λ of 1.01 (SD = 0.01). Thus, sex and PC1 were used as covariates in all MGA tests performed in the study.

## Results

### SNP Association Testing of GGE Candidate Gene Homologs

After realignment and recalibration of the base quality scores of the baboon WGS data, a total of 46,947,610 variants, both SNPs and indels, were successfully called. These variants were then filtered for various QC thresholds, targeting SNPs within known genic regions, yielding 5,634,214 SNPs for 54 case-control animals. For this subset, variant confidence scores normalized by sequence coverage (QualByDepth or QD) range from 2.0 to 42.9, with a mean of 17.5 and median of 16.0 (SD = 6.0). Most (∼79%) of the variants are intronic, with a transition-to-transversion (Ts/Tv) ratio of 2.48. Over 25 million effects on gene products were identified with SnpEff, with 110,771 (0.4%) and 2,394 (0.009%) characterized as “MODERATE” and “HIGH,” respectively, including ones arising from missense and nonsense mutations and splice site variants.

**FIGURE 1:**
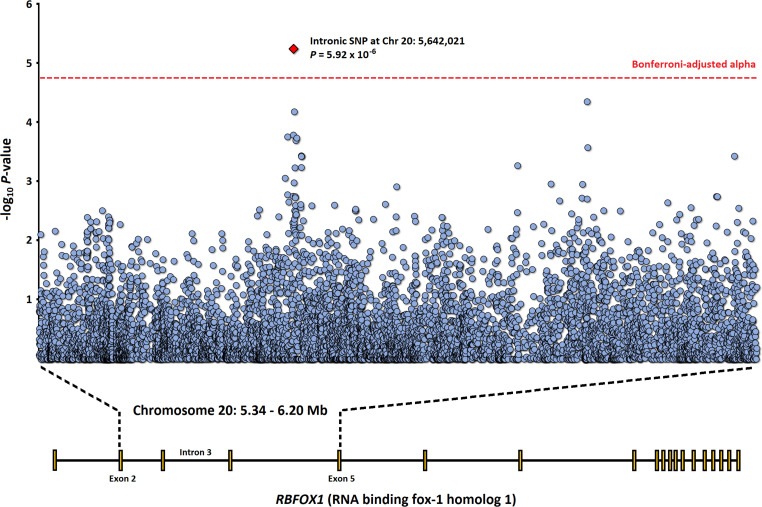
Manhattan plot for part of *RBFOX1* (chromosome 20: 5.34–6.20 Mb), covering exons 2, 3, 4 and 5, and introns 2, 3, and 4. The top SNP association result is shown as a red diamond.

Using a maximum likelihood, variance components approach to our genetic analyses, heritability of epileptic seizure in the baboon cohort was estimated at 0.77 (SE = 0.73; *p* = 0.07). Targeting baboon homologs of genes implicated in GGE and related epilepsies in humans, 20,881 QC filtered SNPs from 19 genes were tested for association with epileptic seizure (see Table 1 and Supplementary Table 1; λ = 1.10). The effective number of independent SNPs tested for association (i.e., accounting for LD between variants) was estimated at 2,686 using the Li & Ji method, (Li and Ji, 2005) which was used in the adjustment of *p*-values for multiple testing *via* the conservative Bonferroni method. The top association result is for a common intronic SNP in the gene *RBFOX1* [chromosome 20 (NC_018171), base pair (bp) position 5,642,021 in intron 3 (ENSPANT00000033712.2); minor allele frequency (MAF) = 0.48], with an estimated β coefficient of 1.37 (SE = 0.30) and Bonferroni-corrected *p* = 0.016 (raw *p* = 5.92 × 10^–6^; see Figure 1). The SNP was successfully genotyped for 51 of the 54 animals, with genotype ratios (AA:AG:GG) of 0:3:12 for healthy controls and 17:12:7 for cases of epileptic seizure, revealing a significant enrichment of the A allele among cases (29 carriers; AF = 0.64) relative to controls (three carriers, all heterozygotes; AF = 0.1). The QC sequencing metrics for the called SNP appear satisfactory, including: QD = 31.3; average genotype quality score (GQ) = 32.3; and approximately 5.9 × depth of coverage.

**TABLE 1:** Top ten candidate genes based on SNP association results for epileptic seizure in baboons.

						Epilepsy seizure	
Gene	Chr	Position (bp)	MA	MAF	Variant type	Beta (SE)	*p*-Value
*RBFOX1*	20	5,642,021	A	0.48	Intron	1.37 (0.30)	5.92 × 10^–6^*
*GABRB3*	7	2,517,399	G	0.10	Intron	5.64 (1.58)	3.67 × 10^–4^
*SCN1A*	12	51,750,877	A	0.22	Intron	3.94 (1.24)	1.50 × 10^–3^
*GABRG2*	6	156,497,930	C	0.09	Intron	1.42 (0.45)	1.73 × 10^–3^
*CASR*	2	41,913,861	A	0.08	Intron	4.65 (1.52)	2.29 × 10^–3^
*CACNA1A*	19	12,102,543	T	0.19	Synonymous	3.56 (1.19)	2.84 × 10^–3^
*GABRA1*	6	156,221,558	C	0.15	Intron	3.54 (1.25)	3.42 × 10^–3^
*CHRNA7*	7	6,949,236	C	0.09	Intron	5.19 (1.77)	3.43 × 10^–3^
*BRD2*	4	32,316,440	G	0.03	Upstream	4.38 (1.50)	3.49 × 10^–3^
*ICK*	4	52,249,073	A	0.09	Intron	4.78 (1.68)	4.55 × 10^–3^

The table lists the top ten gene homologs for human candidate genes for GGE and related epilepsies based on their top SNP association results for epileptic seizure in baboons. SNP positions relative to reference P.h. anubis genome assembly Panu_3.0 (NCBI BioProject PRJNA54005). Variant type determined with the annotation tool SnpEff. *Significant after Bonferroni correction for multiple testing (α = 0.05/2,686 = 1.86 × 10^–5^).

### Genome-Wide Association Testing of Protein-Altering Variants

Given the limited sample size, any comprehensive examination of the millions of called SNPs would be severely underpowered. To achieve a genome-wide perspective of the WGS data, protein-altering SNPs with predicted deleterious effects (*n* = 36,169) were selected for a second round of association testing. It is likely that many of the genetic determinants that underlie seizures in baboons impact protein structure, and thus, targeting this mutation class may allow for broader, functionally meaningful association patterns to emerge. The top association (see Table 2 and Supplementary Table 2; λ = 1.07) is for a missense variant (p.Glu2066Gln) in the gene *SYNE1* (β = 4.35, SE = 0.97; *p* = 7.52 × 10^–6^), which is statistically non-significant after multiple-testing correction. Although the gene has no reported links to epilepsy, *SYNE1* has been identified as the causal gene underlying spinocerebellar ataxia 1 (Gros-Louis et al., 2007).

**TABLE 2:** Top ten genome-wide association results for protein-altering SNPs.

						Epilepsy seizure	
Gene	Chr	Position (bp)	MA	MAF	Variant type	Beta (SE)	*p*-Value
*SYNE1*	4	110,510,700	C	0.06	Missense	4.35 (0.97)	7.52 × 10^–6^
*PBXIP1*	1	125,308,470	G	0.32	Missense	4.09 (0.96)	2.02 × 10^–5^
*SYCP1*	1	112,209,853	A	0.18	Missense	5.63 (1.33)	2.21 × 10^–5^
*LOC108584956*	3	43,438,092	A	0.06	Missense	5.93 (1.42)	3.02 × 10^–5^
*MLLT6*	16	45,549,650	G	0.20	Missense	5.61 (1.38)	4.51 × 10^–5^
*ZNF692*	1	217,379,527	G	0.24	Missense	5.44 (1.33)	4.61 × 10^–5^
*GLYAT*	14	14,601,373	G	0.42	Missense	1.37 (0.34)	5.16 × 10^–5^
*RRM2B*	8	97,957,365	T	0.44	Missense	1.15 (0.30)	1.04 × 10^–4^
*UHRF1BP1*	4	34,215,535	A	0.07	Missense	5.31 (1.39)	1.31 × 10^–4^
*SELENOP*	6	42,584,897	C	0.19	Missense	5.02 (1.34)	1.82 × 10^–4^

*The table lists the top ten SNP association results for epilepsy seizure in baboons for genome-wide variants with deleterious effects on gene products (n = 36,169), as predicted by SnpEff (variants deemed as having either “moderate” or “high” deleterious effect). SNP positions relative to reference P.h. anubis genome assembly Panu_3.0 (NCBI BioProject PRJNA54005). Mutation types analyzed included missense, nonsense, stop codon lost, start codon lost, splice acceptor, and splice donor variants.*

**FIGURE 2:**
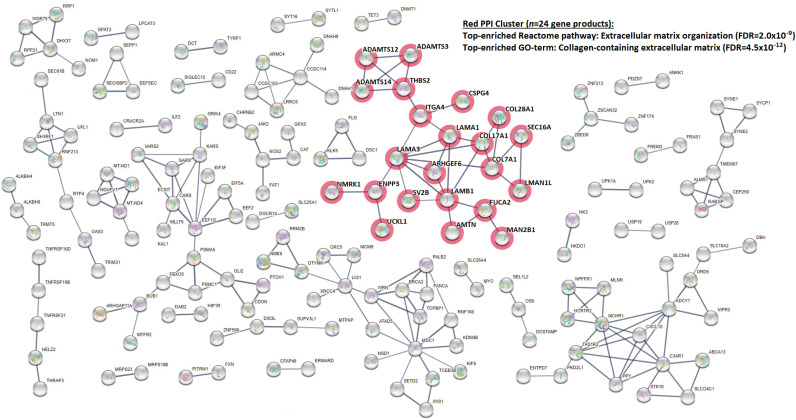
PPI network for protein-altering SNPs with association *p*-values less than 0.01 for epileptic seizure. The overall network includes 34 clusters, ranging in size from 2 to 22 nodes. The largest cluster is highlighted in red, with the top-enriched reactome pathway and GO-term listed. The analyses were conducted for 441 genes with human homolog counterparts with the online program STRING v. 11.0.

For SNP associations with *p*-values less than 0.01 (*n* = 610 SNPs), representing 441 different genes, PPI relationships were examined with STRING v. 11.0 (see Figure 2). Restricted to curated interaction scores with high confidence levels (≥ 0.7), 34 PPI clusters were generated, ranging in size from 2 to 22 nodes, comprising a total of 211 edges, with 264 of the input genes isolated from the network (i.e., unconnected). Given the number of genes examined, the expected number of edges is 172, which yielded a PPI enrichment *p*-value of 2.31 × 10^–3^, thus suggesting broad patterns of biomolecular connectivity among the gene products impacted by these variants. Enrichment analyses were performed on the PPI network (including unconnected nodes), with the following top-enriched GO-terms (FDR = 0.0062): GO:0062023 “Collagen-containing extracellular matrix” (13 of 144 genes belonging to the GO-term category were counted); GO:0005929 “Cilium” (31/570 count); and GO:0005604 “Basement membrane” (11/91 count). Reactome pathways were also tested, but none achieved statistical significance. Narrowing the enrichment analysis to the largest connected cluster in the network, color-coded in Figure 2 as red (*n* = 22 nodes), the top-enriched GO-term and Reactome pathway are GO:0062023 “Collagen-containing extracellular matrix” (9/144 count; FDR = 4.48 × 10^–12^) and HSA-1474244 “Extracellular matrix organization” (9/298 count; FDR = 2.01 × 10^–9^), respectively.

**FIGURE 3:**
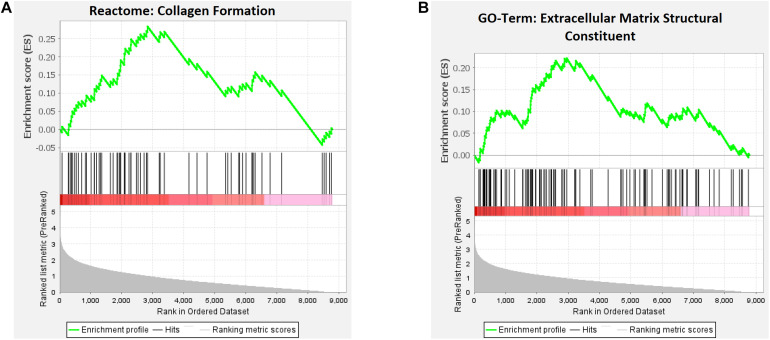
Top-enriched reactome pathway **(A**) and GO-term **(B)** for genes pre-ranked by their top SNP association *p*-value for epileptic seizure among protein-altering variants. The analyses were performed on a total of 8,802 genes with human homologs with the program GSEA v. 4.1. Computation of the running enrichment score was unweighted, with “hits” representing the pre-ranked genes belonging to the curated gene set.

GSEA was also performed on the entire set of protein-altering SNPs, with each of the impacted genes (*n* = 8,802 with human homologs) pre-ranked based on the intragenic SNP with the best association result (and thus not relying on an arbitrarily set *p*-value threshold that partitions the association results). Three gene sets from the Reactome pathways and GO-terms (Table 3) were found to exhibit significant positive enrichment (i.e., overrepresentation among highly ranked genes). Similar to the PPI network, enriched gene sets relate to collagen and the extracellular matrix (ECM). The enriched Reactome pathway is HSA-1474290 “Collagen formation” (FDR = 0.017; see Figure 3A), with 63 genes in the pathway observed in the pre-ranked list, with the top-ranked gene being *ADAMTS3* (73rd gene; max enrichment score (ES) at 2,832nd gene). For the GO-terms, significant enrichment was detected for a pair of gene sets related to the ECM, with the top result belonging to GO:0005201 “Extracellular matrix structural constituent” (FDR = 0.0072; see Figure 3B), with 110 genes in the pre-ranked list, with the top-ranked gene being *MXRA5* (146th gene; max ES at 2,955th gene).

**TABLE 3:** Gene set enrichment analyses (GSEA) of association results for protein-damaging SNPs.

Gene set	Size	NES	FDR *q*-value
Reactome pathways			
Collagen formation	63	2.62	1.67 × 10^–2^
GO-terms			
ECM structural constituent	110	2.71	7.16 × 10^–3^
ECM structural constituent conferring tensile strength	32	2.48	2.88 × 10^–2^

*The table lists significant GSEA results for Reactome pathways and GO-terms (MSigDB v. 7.2). The gene list input is based on the association results for epileptic seizure in baboons for 36,169 protein-damaging SNPs, pre-ranked according to the top associations in each gene (negative logarithmic transformation of unadjusted p-values). Size is the number of pathway/GO-term genes observed in the gene list. NES is the normalized enrichment score, which represents the degree to which a gene set is overrepresented at the top (or bottom) of the pre-ranked list of genes.*

## Discussion

In this study, genetic association testing was performed for epileptic seizure in a pedigreed baboon colony using available WGS data from the NCBI SRA database. Targeting homologs of genes previously implicated in human epilepsy, we observed a significant association for an intronic SNP in the gene *RBFOX1*, encoding an RNA-binding protein that regulates splicing, including transcripts of other epilepsy candidate genes (e.g., *GABRG2*, *KCNQ2*, *SCN8A*, *SLC12A5*, and *SYN1*, among others), and plays a key role in neuronal excitation in the mammalian brain (Gehman et al., 2011). A more agnostic approach was also used in our analyses, as protein-altering SNPs were identified throughout the baboon genome and tested for association with epilepsy status. Although no significant associations were observed, genes involved in the collagen-containing ECM showed significant overrepresentation among top results. To the best of our knowledge, these findings represent the first characterization of potential variants underlying the genetic architecture of epilepsy in baboons, pointing to commonalities with what is known about the human genetic etiology, thus suggesting the suitability of baboons as an animal model for the disorder.

The *RBFOX1* gene has repeatedly been linked to epilepsy, with the earliest reports based on *de novo* structural gene variants observed in patients comorbid for autism, (Martin et al., 2007) intellectual disability, (Bhalla et al., 2004) or pontocerebellar hypoplasia (Gallant et al., 2011). These findings were followed up by a series of association studies by Lal et al. that targeted exon-disrupting microdeletions in *RBFOX1*, revealing a significant excess among GGE patients compared to population controls, (Lal et al., 2013b) as well as among cases of Rolandic epilepsy (Lal et al., 2013a) and sporadic focal epilepsy, (Lal et al., 2015) suggesting that *RBFOX1* deletions are involved in a wide spectrum of both focal and generalized epilepsies. In mice, brain-specific *Rbfox1* knockouts have displayed spontaneous seizures and heightened epileptogenic response to kainic acid (Gehman et al., 2011). Although the pathophysiological mechanisms of RBFOX1 remain to be clarified, more recent *in vivo* and *in vitro* studies have yielded insights, including regulation of cerebral cortex development, (Hamada et al., 2015) changes to transcriptomic expression and splicing patterns of neuronal genes, (Fogel et al., 2012) and crosstalk with microRNA miR-129-5p that impacts homeostatic downscaling of excitatory synapses (Rajman et al., 2017).

As one of the longest genes in the human genome, *RBFOX1* has an extended 5′ region that harbors several transcription start sites at varying first exons, enabling complex isoform versatility to produce differential patterns of subcellular and tissue-specific localization (Conboy, 2017). The key regulatory feature of the RBFOX1 protein, however, is located in the downstream coding region, a single RNA recognition motif (RRM) that preferentially binds the sequence (U)GCAUG in pre-mRNA introns, mRNA 3′ UTRs, and microRNA hairpins. Alternative splicing modulates the inclusion of the various exons in this region, generating active and inactive forms of RRM, as well as directing isoform localization. Overall, the gene is highly conserved in most primates, including its homolog in *P.h. anubis* that is approximately 92.4% identical to its human counterpart, with a Gene Order Conservation score of 100 (Ensembl Release 102 data).

For the significant *RBFOX1* association reported here, the SNP is positioned in intron 3 (of 19 in total), which is part of the extended 5′ region described above. In the human genome (GRCh38), the SNP and its flanking 50 bp region aligns within *RBFOX1* at chromosome 16: 5,767,827–5,767,900 bp (cytogenetic band 16p13.3) *via* the UCSC BLAST-like alignment tool (score = 58; span = 74 bp; identity = 89.2%), and appears to have limited functional and clinical relevance, besides a few nearby *cis*-regulatory elements (DNase-H3K4me3, proximal enhancer-like signatures). The intron itself harbors SNPs listed in the NHGRI-EBI GWAS catalog *(p* < 1.0 × 10^–5^), with perhaps the most noteworthy being rs13332522, a variant significantly associated with ADHD-related variation in intracranial volume (Klein et al., 2019). For GWAS of epilepsy, the recent mega-analysis by ILAE is the most substantive dataset to-date, (The International League Against Epilepsy Consortium on Complex Epilepsies, 2018) revealing 16 genome-wide significant loci. Although this does not include *RBFOX1*, a suggestive multi-variant association signal is evident in intron 7 for GGE, with a peak at rs11639540 *(p* = 1.84 × 10^–6^).

Further, our analysis of genome-wide protein-damaging variants in baboon indicates a potential etiological role for collagen-containing ECM, with links to epilepsy reported in literature. ECM has long been known to regulate various aspects of neural development, providing structural support (e.g., basement membrane scaffolded by collagen fibrils), and stimulating various pathways that drive progenitor proliferation, neuronal differentiation and migration, and synaptogenesis (Long and Huttner, 2019). During epileptogenesis, the perineuronal net (PNN), a condensed ECM structure that envelops fast-spiking interneurons and that is responsible for synaptic plasticity and excitation-inhibition stabilization, has shown evidence of degradation by endogenous proteases, resulting in impaired GABAergic inhibition (Tewari et al., 2018). Matrix metalloproteinase-9 (MMP-9) is one such PNN protease, which has exhibited increased mRNA and protein levels after seizure in both animal models and patients, including generalized tonic–clonic events, (Cudna et al., 2017) with inhibitors viewed as potential therapeutic drug targets (Broekaart et al., 2021).

These findings provide a compelling first glimpse into the genetic underpinnings of epilepsy in baboons, targeting common, less penetrant risk variants in the allele frequency spectrum. A significant burden of rare, deleterious microdeletions at recurrent hotspots among neurodevelopmental genes and subject to negative selection has been reported in GGE patients, including *RBFOX1* as noted above, and thus, analyses of genome-wide indels in the baboon sequence data, including the delineation of haplotype structures at key loci, could be highly informative. Moreover, the sample size of this preliminary investigation is relatively small and could be boosted by additional baboons from the pedigreed colony, providing the requisite power to detect novel risk variants that are rarer and of smaller effect size. With its extensive archived WGS data and veterinary records, the SNPRC colony could provide a unique resource for epilepsy research, namely, a primate model with a shared genetic etiology, which can garner new insights and lead to much-needed alternative approaches to therapeutic treatment.

## Data Availability Statement

The original contributions presented in the study are included in the article/Supplementary Material, further inquiries can be directed to the corresponding author/s.

## Ethics Statement

The animal study was reviewed and approved by IACUC of the University of Texas Health Science Center at San Antonio and the Texas Biomedical Research Institute.

## Author Contributions

MK, MC, and CS devised the project. MK processed the whole-genome sequence data and performed the genetic analyses. ML, KK, and CS conducted the retrospective survey of veterinary records. CS performed the diagnoses of epilepsy in the baboon colony. MK and CS took the lead in writing the manuscript. MC, LB, and HG provided critical feedback that helped shape the analysis and manuscript. All authors contributed to the article and approved the submitted version.

## Conflict of Interest

The authors declare that the research was conducted in the absence of any commercial or financial relationships that could be construed as a potential conflict of interest.

## Publisher’s Note

All claims expressed in this article are solely those of the authors and do not necessarily represent those of their affiliated organizations, or those of the publisher, the editors and the reviewers. Any product that may be evaluated in this article, or claim that may be made by its manufacturer, is not guaranteed or endorsed by the publisher.

## Footnotes

1
http://broadinstitute.github.io/picard

2
https://gatk.broadinstitute.org/hc/en-us/articles/360035531112–How-to-Filter-variants-either-with-VQSR-or-by-hard-filtering
