# Supplementary figures and images for: Whole Genome Sequence Data From Captive Baboons Implicate RBFOX1 in Epileptic Seizure Risk
Source: Front Genet. 2021 Aug 20;12:714282. doi: 10.3389/fgene.2021.714282 (PMC8417722; doi:10.3389/fgene.2021.714282)

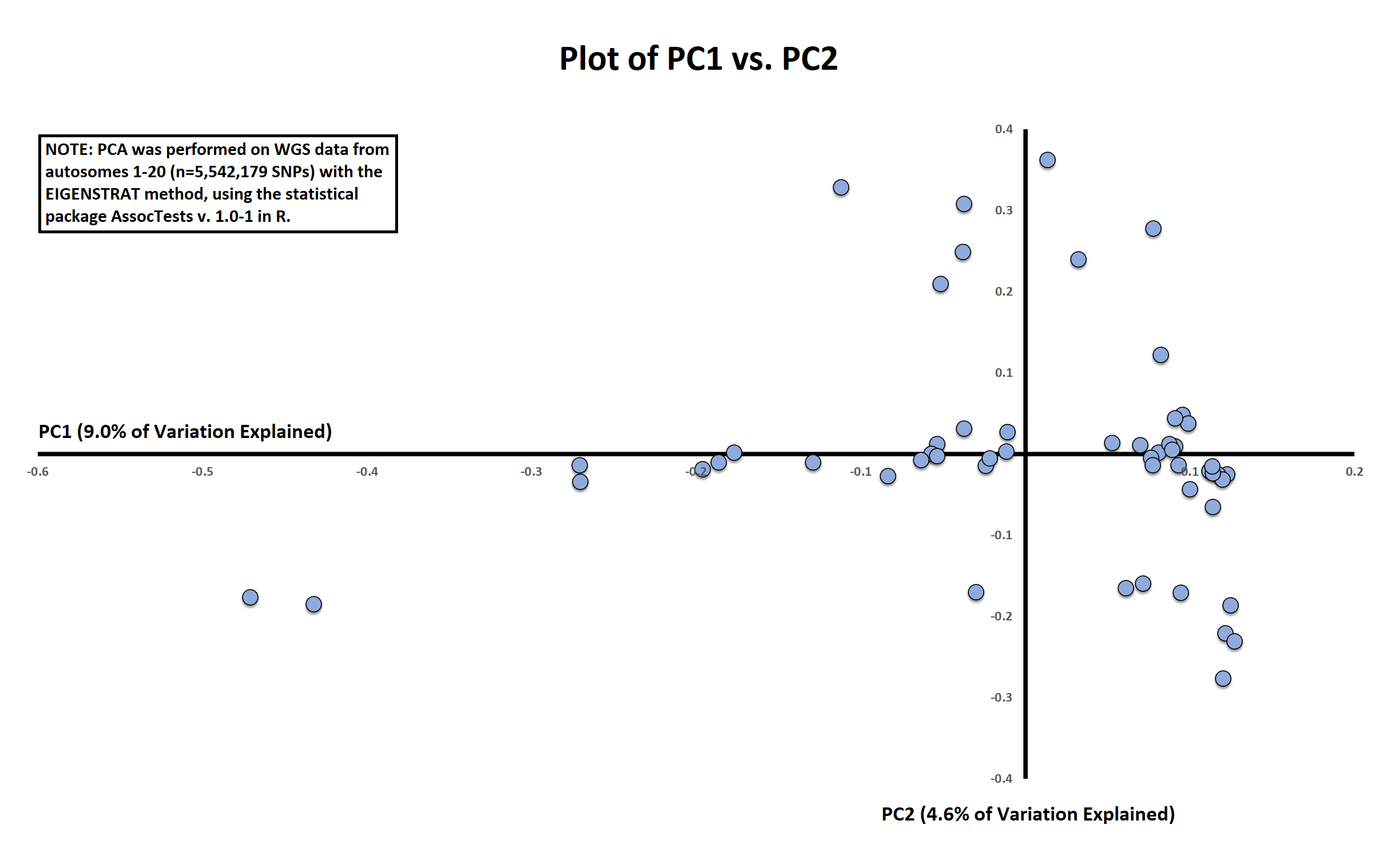

Supplement: Supplementary file 1 [file Image_1.PNG]

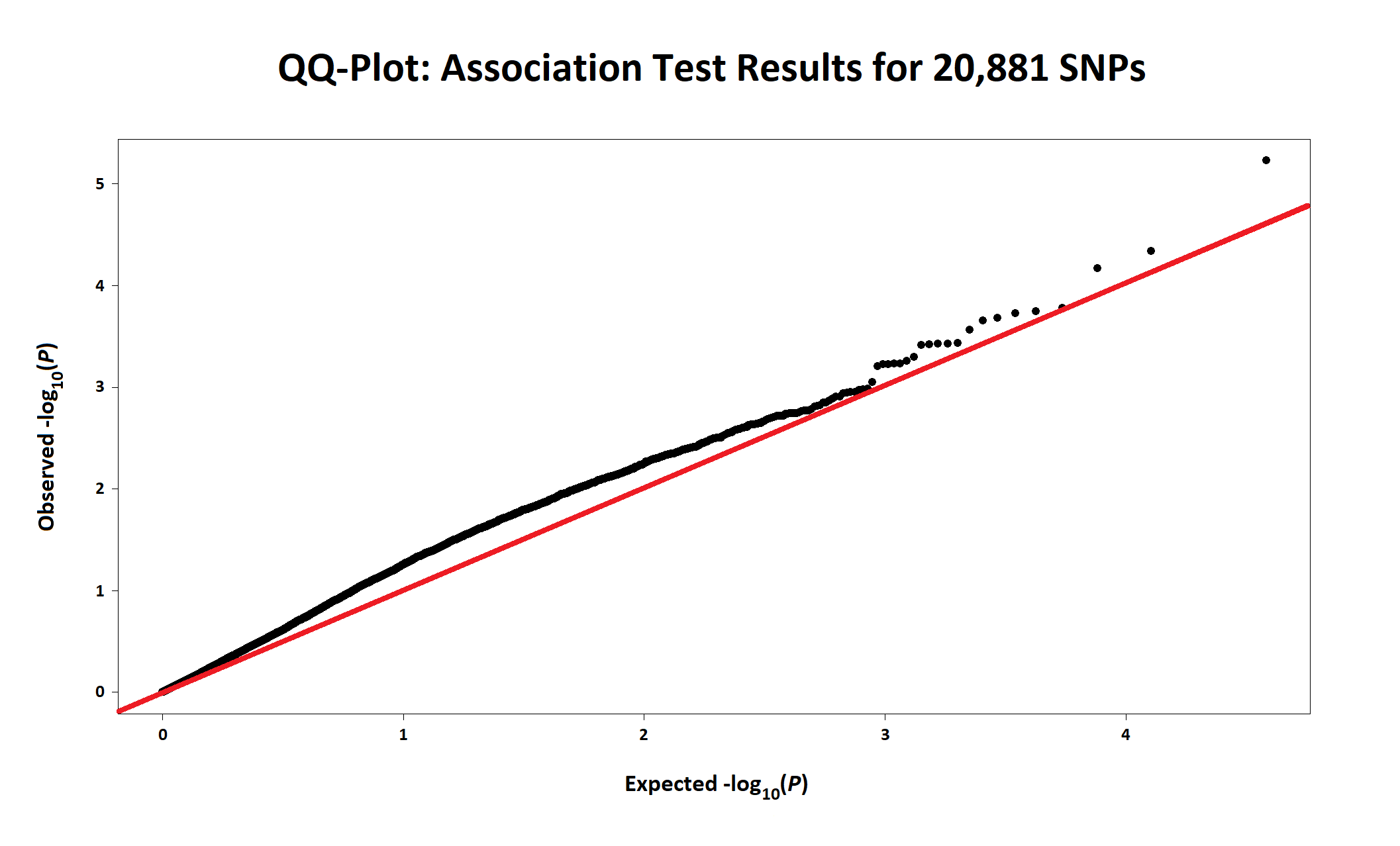

Supplement: Supplementary file 2 [file Image_2.PNG]
